# Risk of delayed bleeding after hemorrhoidectomy

**DOI:** 10.1007/s00384-018-3176-6

**Published:** 2018-10-24

**Authors:** Ko-Chao Lee, Chia-Cheng Liu, Wan-Hsiang Hu, Chien-Chang Lu, Shung-Eing Lin, Hong-Hwa Chen

**Affiliations:** 1 Division of Colorectal Surgery, Department of Surgery, Chang Gung Memorial Hospital - Kaohsiung Medical Center, Chang Gung University College of Medicine, No. 123, DAPI Rd. Niaosng Dist, Kaohsiung City, 83301 Taiwan; 2 Department of Surgery, Pingtung Christian Hospital, Pingtung, Taiwan

**Keywords:** Hemorrhoidectomy, Ferguson procedure, LigaSure procedure, Delayed post-hemorrhoidectomy bleeding, Risk factors

## Abstract

**Purpose:**

Delayed post-hemorrhoidectomy bleeding (DPHB) is a rare but serious complication. We investigated the incidence and risk factors of DPHB in patients undergoing hemorrhoidectomy using the LigaSure device or the Ferguson procedure.

**Methods:**

This retrospective study included 382 consecutive patients with symptomatic grades II to IV hemorrhoids who received either LigaSure (184 patients) or Ferguson (198 patients) hemorrhoidectomy procedures. Thirty-two patients who experienced DPHB after discharge were followed up.

**Results:**

Significantly fewer Ferguson group patients had DPHB compared to the LigaSure group (5.1% vs. 11.9%; *P* = 0.015). In the overall population, the risk of DPHB was higher in (1) males compared to that of females (OR = 3.39; 95% CI 1.50–7.69, *P* = 0.003); (2) in the LigaSure group compared to the Ferguson group (OR = 2.77; 95% CI 1.23–6.24, *P* = 0.01); and (3) in patients with constipation (OR = 6.59; 95% CI 2.73–15.89, *P* < 0.0001). Males in the LigaSure group had a significantly higher rate of delayed bleeding than those in the Ferguson group (20% vs. 5.8%, *P* = 0.004); no significant differences were found in females (4.9% vs. 4.5%, *P* = 0.878). Subgroup analysis showed that in males, risk of DPHB increased significantly with postoperative constipation (OR = 4.73, 95% CI 1.45–15.43, *P* = 0.010) and the LigaSure procedure (OR = 3.99, 95% CI 1.37–11.62, *P* = 0.011). In females, the risk of DPHB was significantly associated with postoperative constipation (OR = 8.80, 95% CI 2.24–34.54, *P* = 0.002).

**Conclusions:**

The LigaSure procedure and constipation are independent risk factors for DPHB in patients undergoing hemorrhoidectomy and can be used as predictors of outcome.

## Introduction

Hemorrhoidal disease is a common anorectal disorder characterized by the symptomatic enlargement and distal displacement of the hemorrhoids, which are the normal anal cushions [1, 2]. The main symptoms of hemorrhoidal disease include rectal bleeding, prolapse, pain, thrombosis, mucus discharge, and pruritus [3]. Hemorrhoids are classified based on their location and the degree of prolapse. Internal hemorrhoids originate from the internal hemorrhoidal plexus above the dentate line and are classified by the degree of prolapse of the anal canal. External hemorrhoids originate from the external hemorrhoidal plexus below the dentate line and are classified as acute (hemorrhoidal thrombosis) or chronic (anal skin tags) [4]. Although the etiology is not completely understood, constipation and prolonged straining resulting in hard stool and increased intra-abdominal pressure are thought to obstruct venous return, and cause engorgement of the hemorrhoidal plexus [5].

Clinical management of hemorrhoidal disease is based on severity. While conservative strategies are used for patients with grades I or II disease [6, 7], radical surgery is recommended for patients with grades III or IV hemorrhoids. Open hemorrhoidectomy (Milligan and Morgan procedure) and closed hemorrhoidectomy (Ferguson procedure) are the most commonly used surgical techniques for excision of the hemorrhoidal cushions and remain the gold standard of surgical treatment [8]. However, these techniques are associated with postoperative pain and blood loss [9]. The LigaSure vessel sealing system was developed to reduce anal spasm and pain after hemorrhoidectomy. It uses a combination of pressure and electrical energy to ensure complete coagulation of vessels up to 7 mm in diameter, with minimal thermal spread and tissue charring [10, 11]. Studies that compared the outcomes of patients who underwent the Ferguson procedure to the outcomes of those who underwent hemorrhoidectomy using the LigaSure system reported a number of short-term benefits associated with the LigaSure procedure, including reduced intraoperative blood loss, reduced operating time, and reduced postoperative pain [9, 12–15].

Delayed post-hemorrhoidectomy bleeding (DPHB) is a rare but serious complication after hemorrhoidectomy [16]. The incidence of delayed postoperative hemorrhage has been reported to be 0.9–10% [17, 18]. Some data suggest that DPHB is linked to risk factors such as the surgical procedure, infection, defecation with excessive straining, and number of piles [19–21]. Interestingly, a study that evaluated 45 patients with DPHB reported that male gender and individual surgeons were independent risk factors for DPHB [17]. There was no significant difference in the occurrence of DPHB between patients who underwent a closed or open hemorrhoidectomy [22], or between the conventional hemorrhoidectomy and using the LigaSure system [9].

In this study, we investigated the incidence of DPHB following hemorrhoidectomies performed using the LigaSure system and the Ferguson procedure. We also investigated the risk factors associated with DPHB.

## Patients and methods

This retrospective study enrolled a total of 382 consecutive patients with symptomatic grades II to IV hemorrhoids who underwent either hemorrhoidectomy using the LigaSure system (184 patients; LigaSure group) or the Ferguson procedure (198 patients; Ferguson group) at the Kaohsiung Chang Gung Memorial Hospital between January 2009 and December 2009. Patients were assigned to either procedure at the surgeon’s discretion. Patients who received other procedures in addition to hemorrhoidectomy (*n* = 47) and patients with no data regarding number of excised hemorrhoids (*n* = 2) were excluded from the study. Patients on anticoagulant medication or aspirin were asked to stop their medication 7 days prior to surgery. Patients were admitted to the Surgery Ward on the morning of the procedure and discharged the next day, except in the event of a postoperative complication.

### Definitions

Constipation was defined as bowel movements that were infrequent or hard to pass. Since it is difficult to feel solid stool stuck in the rectum during an anal examination, a finding of constipation was based on patients’ self-report of difficulty in passing stool. Stool impaction was defined as development of solid, immobile feces in the rectum, causing discomfort, an urge to defecate, easy wound bleeding, and anal contraction. These cases of constipation with impaction are treated at our hospital with oil retention and cleansing enemas to allow discharge of the stool stuck at the end of the rectum.

### Procedures

The same physicians from our surgical group performed all hemorrhoidectomies, including both Ferguson and LigaSure procedures. All procedures were carried out under general anesthesia with intravenous propofol and a laryngeal mask airway. The procedure was carried out with the patient in the modified Sim’s position (left lateral decubitus position with both knees and thighs drawn upward toward the chest). The initial steps in hemorrhoidectomy using the LigaSure system and the Ferguson hemorrhoidectomy were identical and included (1) manual anal sphincter stretching up to four fingers, (2) delivery of hemorrhoidal masses with artery forceps, one being applied at the base of hemorrhoid, the other at the apex, (3) skin incision at the base of the hemorrhoids and submucosal dissection to lift the hemorrhoid mass off the internal sphincter by monopolar diathermy.

Following these steps, the Ferguson method involved transfixing the hemorrhoid pedicle with 2/0 chronic catgut sutures and opposing the mucosal edges of the defect with 2/0 chromic catgut. In the LigaSure method, the jaws of the handset were applied on the pedicle, and the instrument activated by the foot paddle. A computer-controlled feedback loop automatically stopped the flow of energy when coagulation of the vessels and mucosa was achieved. The hemorrhoid mass was excised with scissors by cutting across the coagulated tissue seal. No sutures were applied as the LigaSure device also achieved mucosal fusion. Anal canal packing was not routinely done except when complete homeostasis was doubtful.

Patients were encouraged to take a Sitz bath on the morning after the surgery, and laxatives were not routinely prescribed except for patients with a history of constipation. After discharge, all patients receiving hemorrhoidectomy were scheduled for at least two follow-up visits. The first visit was within 1 week after surgery, and the second visit was 2~4 weeks after surgery. Patients with delayed postoperative hemorrhage occurring after discharge were followed up longer (37.0 ± 40.7 [range 1–323] days). Only patients who left the hospital after hemorrhoid surgery, bled to the amount of at least a bowl (200 ml), and went to the emergency room were included as DPHB in the analysis.

The study protocol was approved by the IRB of Kaohsiung Chang Gung Memorial Hospital.

### Statistical analysis

Mean and standard deviation were used to present continuous variables. Number and percentage were computed for categorical variables. Significance of differences between patients receiving different surgeries was determined using the independent two-sample *t* test and chi-squared test. When 20% of cells had an expected value < 5, the chi-square test was replaced with the Fisher exact test. Odds ratio (OR) and 95% confidence intervals (CIs) were estimated by logistic regression to quantify the strength of association between factors and postoperative bleeding. Variables with *P* < 0.05 were then used to establish the multivariable regression model. *P* values < 0.05 were considered statistically significant. All statistical analyses were two-sided and performed with PASW software (version 22, IBM Corp., Armonk, NY).

## Results

### Participants’ characteristics

The demographics and clinical characteristics of the study participants are summarized in Table 1. No significant differences were found in age, gender, or hematological index between the two groups.

**Table 1:** Demographics and clinical characteristics of 382 study participants

	LigaSure (*n* = 184)	Ferguson (*n* = 198)	*P*
Age (years)	48.5 ± 14.0	46.6 ± 12.8	0.167
Male	83 (45.1)	87 (43.9)	0.818
Wounds status			
< 3 wounds	24 (13.0)	44 (22.2)	*0.03*
≥ 3 wounds	160 (86.9)	154 (77.8)	
Vital signs			
Baseline SBP (mmHg)	120.0 ± 13.1	121.2 ± 13.9	0.412
Baseline DBP (mmHg)	72.8 ± 8.6	74.2 ± 9.9	0.204
Baseline pulse (beats/min)	73.7 ± 8.9	74.8 ± 10.9	0.324
Baseline platelet (10^4^/CMM)	242.9 ± 63.9	247.3 ± 56.0	0.514
History of general medical diseases	29 (15.8)	25 (12.6)	0.379
Hospital stay (days)	2.44 ± 2.03	4.06 ± 1.53	*< 0.001*
Comorbidity			
Diabetes	6 (3.3)	7 (3.5)	0.883
Hypertension	19 (10.3)	21 (10.6)	0.929
Cardiovascular	5 (2.7)	1 (0.5)	0.082
End-stage renal disease	2 (1.1)	0 (0)	0.231
Anemia	1 (0.5)	8 (4.0)	*0.04*
Stroke	2 (1.1)	1 (0.5)	0.611
Hepatitis B or C	1 (0.5)	15 (7.6)	*0.001*
Asthma	1 (0.5)	6 (3.0)	0.123
Long-term control medications	25 (13.6)	19 (9.6)	0.222
Outcomes			
Postoperative bleeding	22 (11.9)	10 (5.1)	*0.015*
Time to postoperative bleeding (days^a^)	7.2 ± 4.4	7.8 ± 3.9	0.707

Continuous variables are presented as mean ± standard deviation and categorical variables are shown as frequency (%)

Italic value indicates significant difference between two groups, *P* < 0.05

^a^Only data from 32 patients with delayed postoperative bleeding occurring after discharge was used for analysis

No significant differences were found between the LigaSure and Ferguson groups in the incidence of comorbidities such as diabetes, hypertension, cardiovascular disease, end-stage renal disease, stroke, or asthma. However, patients in the Ferguson group had a significantly higher incidence of anemia (4.0% vs. 0.5%, *P* = 0.04) and hepatitis B or C compared to patients in the LigaSure group (7.6% vs. 0.5%, *P* = 0.001). The Ferguson group had a significantly lower percentage of patients with three or more excised hemorrhoids compared to the LigaSure group (154 patients or 77.8% vs. 160 patients or 86.9%, respectively; *P* = 0.03). The Ferguson group also had a significantly lower percentage of patients with delayed postoperative bleeding occurring after discharge compared to the LigaSure group (10 patients or 5.1% vs. 22 patients or 11.9%, respectively; *P* = 0.015). Two patients in the Ferguson group and two patients in the LigaSure group received further surgery to stop bleeding. Although the time interval between surgery and bleeding in the Ferguson group was slightly longer (7.8 ± 3.9 days vs. 7.2 ± 4.4 days) compared to that in the LigaSure group, this was not statistically significant. In the LigaSure group, 63.6% of cases had delayed postoperative bleeding events within 1 week after surgery, compared to 40.0% in the Ferguson group during the same period (Table 1).

### Risk factors for postoperative bleeding

A total of 350 patients had no delayed postoperative bleeding after discharge, while delayed postoperative bleeding occurred in 32 patients after discharge. Univariate analysis showed that male gender, LigaSure procedure, stool impaction, and constipation were significant risk factors for delayed postoperative bleeding (Table 2). Results from the multivariate analysis showed that male gender, LigaSure procedure, and constipation were independent risk factors for delayed postoperative bleeding. Risk of delayed postoperative bleeding was 3.39-fold higher in males compared to that of females (95% CI 1.50–7.69, *P* = 0.003) and 2.77-fold higher in the LigaSure group than in the Ferguson group (95% CI 1.23–6.24, *P* = 0.01). Patients with constipation had an OR of 6.59 (95% CI 2.73–15.89, *P* < 0.0001) compared to those without. No significant differences were found in risk of delayed postoperative bleeding between patients with fewer than three excised hemorrhoids and those with three or more excised hemorrhoids (Table 2).

**Table 2:** Predictors of postoperative bleeding

	Univariate analysis		Multivariable analysis	
	OR (95% CI)	*P*	OR (95% CI)	*P*
Age (years)	0.98 (0.965, 1.01)	0.178		
Male	3.00 (1.37, 6.53)	*0.006*	3.39 (1.50, 7.69)	*0.003*
LigaSure	2.55 (1.17–5.55)	*0.018*	2.77 (1.23, 6.24)	*0.01*
Wounds status				
< 3 wounds	1	0.052		
≥ 3 wounds	7.33 (0.98, 54.64)			
Vital signs				
Baseline SBP (mmHg)	1.02 (0.99, 1.05)	0.101		
Baseline DBP (mmHg)	1.03 (0.99, 1.07)	0.150		
Baseline pulse (beats/min)	1.01 (0.98, 1.05)	0.496		
Baseline platelet (10^4^/CMM)	1.00 (0.99, 1.01)	0.676		
Comorbidity				
Diabetes	0.91 (0.11, 7.22)	0.927		
Hypertension	1.67 (0.60, 4.60)	0.324		
Cardiovascular	5.77 (1.02, 32.79)	0.048		
Anemia	1.38 (0.17, 11.39)	0.764		
Stroke	5.62 (0.49, 63.68)	0.164		
Hepatitis B or C	0.72 (0.09, 5.64)	0.755		
Asthma	1.85 (0.22, 15.86)	0.575		
Long-term control medications	1.48 (0.54, 4.06)	0.449		
Postoperative features				
Medication	0.79 (0.29, 2.15)	0.654		
Urine retention	0.99 (0.44, 2.22)	0.985		
Stool impaction	4.26 (1.56, 11.65)	*0.005*		
Constipation	5.03 (2.23, 11.34)	*< 0.001*	6.59 (2.73, 15.89)	*< 0.0001*
Laxative usage	0.69 (0.34, 1.43)	0.317		

*OR*, odds ratio; *CI*, confidence interval

Italic value indicates significant difference, *P* < 0.05

### Men have a higher postoperative bleeding rate after LigaSure hemorrhoidectomy

Since our data showed that males were more prone to delayed postoperative bleeding than females, we investigated effectors of outcomes using subgroup analysis by gender (Table 3). Male patients in the LigaSure group had a significantly higher rate of delayed bleeding than those in the Ferguson group (20% vs. 5.8%, *P* = 0.004), while there was no significant difference in the bleeding rate between females in the two groups (4.9% vs. 4.5%, *P* = 0.878) (data not shown). In females, the risk of delayed postoperative bleeding was significantly associated with postoperative constipation (OR = 8.80, 95% CI 2.24–34.54, *P* = 0.002). In males, the LigaSure procedure (OR = 3.99, 95% CI 1.37–11.62, *P* = 0.011) and postoperative constipation also increased the risk of delayed postoperative bleeding (OR = 4.73, 95% CI 1.45–15.43, *P* = 0.010) (Table 3).

**Table 3:** Predictors of postoperative bleeding in males and females

	Female		Male	
	OR (95% CI)	*P*	OR (95% CI)	*P*
LigaSure	1.65 (0.42, 6.45)	0.470	3.99 (1.37, 11.62)	*0.011*
Postoperative constipation	8.80 (2.24, 34.54)	*0.002*	4.73 (1.45, 15.43)	*0.010*

*OR*, odds ratio; *CI*, confidence interval

Italic value indicates significant difference, *P* < 0.05

## Discussion

This retrospective study compared risk factors for delayed postoperative hemorrhage occurring after discharge in patients who had undergone hemorrhoidectomy using the LigaSure device with those who had undergone the Ferguson procedure. We found that male gender and constipation were independent risk factors of delayed postoperative bleeding. Interestingly, the LigaSure procedure was also a significant risk factor for delayed postoperative hemorrhage. The LigaSure procedure increased the risk of delayed postoperative bleeding more significantly in males than in females.

Hemorrhoidectomy is one of the most common procedures performed in anorectal surgery [23]. Conventional hemorrhoidectomy procedures have been shown to be associated with significant postoperative complications such as tissue trauma, pain, bleeding, and anal stricture [24]. Recent studies reported that patients receiving the LigaSure procedure experienced reduced postoperative pain, reduced tissue damage, and more rapid wound healing compared to conventional hemorrhoidectomy, although there was no significant difference in the overall incidence of complications [13, 25, 26]. A previous meta-analysis of nine randomized controlled trials reported that patients who underwent the LigaSure procedure had a significantly lower operative time and blood loss but had no significant benefit in terms of postoperative pain, or length of hospital stay compared to patients who underwent conventional hemorrhoidectomy [27, 28]. However, other data indicated that patients in the LigaSure group had a shorter operative time and hospital stay compared to patients in the Ferguson group [29]. In the present study, patients in the LigaSure group had a significantly shorter duration of hospital stay compared to patients in the Ferguson group.

DPHB is an important complication of hemorrhoidectomy and can occur between the fourth and eighteenth day after the procedure [30]. There are few studies that have investigated the occurrence and risk factors of DPHB. A meta-analysis of nine studies reported no significant difference in postoperative bleeding between patients with grades II or IV hemorrhoids who either underwent conventional hemorrhoidectomy or the LigaSure procedure [27]. A prospective study of 4880 patients who underwent the Ferguson procedure showed that male gender and surgeon variability were risk factors for DPHB [17], while other studies found that these did not impact the occurrence of DPHB [21, 31], possibly because of the small sample sizes of these studies. A prospective, randomized study of 61 patients who underwent either the Ferguson procedure or the LigaSure procedure found that although the LigaSure procedure reduced operative time and postoperative pain, there was no significant difference in postoperative bleeding between the two groups, possibly due to the slow wound healing process after the LigaSure procedure [13]. A study of 24 patients who underwent the Ferguson procedure reported that there was a greater likelihood of early period hemorrhage (within 3 days of the procedure) at the posterior wall [21]. In the present study, ten patients had delayed bleeding after Ferguson surgery, of which two received further surgery to stop bleeding. Twenty-two patients had delayed bleeding after LigaSure surgery, of which two received further surgery to stop bleeding. Most delayed bleeding after hemorrhoidectomy using the LigaSure device can be stopped through conservative treatment, and only a few patients with severe delayed bleeding need to return to the operating room for further surgical treatment.

Approximately 99% of Taiwanese are covered by the country’s National Health Insurance system. Therefore, even when patients who have postoperative complications are treated in a different hospital from the one where surgery was performed, all medical records are easily accessible. We were therefore able to ascertain whether any of the study patients were treated for delayed bleeding. Our present study found a higher incidence of DPHB compared to previous reports, especially in the LigaSure group. This could possibly be because of the reduced pain reported by a proportion of patients who received follow-up after the LigaSure procedure, which might lead to patient hyperactivity. Indeed, during the follow-up visits, some patients reported that the relative lack of pain after the LigaSure procedure resulted in a quicker return to laborious daily activities or strenuous exercise, which could lead to delayed postoperative bleeding. Although pain status after the two procedures was not reported by all patients at follow-up, previous studies have reported that patients who underwent the LigaSure procedure had shorter procedure durations, less postoperative pain, and returned to work significantly earlier compared to patients who underwent conventional hemorrhoidectomy [15, 32]. We compared the risk factors of DPHB in patients who underwent the Ferguson procedure with patients who underwent the LigaSure procedure. Our data showed that the LigaSure procedure and postoperative constipation were significant independent risk factors of DPHB in males and females. Additionally, males had a higher risk of postoperative bleeding after hemorrhoidectomy using the LigaSure device. We speculate that this could be due to the longer anal canal and the typically higher physical activity levels in male patients in Taiwan.

In both the LigaSure and Ferguson groups, no significant differences were found in delayed postoperative bleeding between patients using a postoperative laxative and those who did not. These data suggest that even after using laxatives to soften the stool after hemorrhoidectomy, postoperative constipation still causes a high risk of delayed postoperative bleeding. Our data also show that, although no significant differences are found in the risk of postoperative bleeding between patients with fewer than three excised hemorrhoids and those with three or more excised hemorrhoids, there was a trend toward delayed bleeding in the case of three or more excised hemorrhoids.

To the best of our knowledge, this is the first study comparing the occurrence of delayed post-hemorrhoidectomy bleeding after using the LigaSure device to that after using the Ferguson procedure. The validity of our conclusions is strengthened by the fact that this study enrolled and analyzed the largest patient pool so far (198 patients in the Ferguson group and 184 patients in the LigaSure group). The most important limitation of this study was that it is a cross-sectional, retrospective, non-randomized analysis. Retrospective study with the reports of multiple surgeons did not allow us to evaluate postoperative pain or return to work because it was not reported for all patients. Another important limitation was the time delay associated with standardization of protocols for surgeons who were initially inexperienced with the LigaSure device. Although most surgeons are familiar with the use of LigaSure for various other types of disease, surgery performed by a surgeon not used to the procedure can be associated with an increased risk of delayed bleeding, and it may have been better to study a cohort in which the patients received the LigaSure procedure after the surgeons had completed their learning curve. Although analyzing the effect of learning curves is outside the scope of the present study, future studies should address this issue. Our data have important implications for the clinical management of hemorrhoidectomy using the LigaSure device in order to improve outcomes in these patients. It will be important to validate our findings in large, multi-center, prospective trials.

## Conclusions

The LigaSure procedure and constipation are independent risk factors for delayed postoperative bleeding in patients undergoing hemorrhoidectomy. Risk of delayed postoperative bleeding is significantly associated with postoperative constipation in females and with the LigaSure procedure and postoperative constipation in males. Identification of risk factors that predict postoperative hemorrhage may help improve the clinical management of patients for whom hemorrhoidectomy has been recommended.
